# Disruption of the Muscle–Brain Axis in Ageing: Role of Myokines in Sarcopenia and Neurodegeneration

**DOI:** 10.3390/cells15201910

**Published:** 2026-10-20

**Authors:** Víctor Agustín Morales Molina, Cecibel Nicole Ludeña Salinas, Celina Andrade Vásquez, Andri Matos, Diego Aldeán Riofrío

**Affiliations:** 1 Health Sciences Department, School of Medicine, Universidad Técnica Particular de Loja, Loja 1101608, Ecuador; vamorales7@utpl.edu.ec (V.A.M.M.); cnludena@utpl.edu.ec (C.N.L.S.); dmaldean02@utpl.edu.ec (D.A.R.); 2 Allied Health Department, School of Allied Health, Eastwick College, Ramsey, NJ 07446, USA; andry_9@hotmail.com

**Keywords:** myokines, skeletal muscle–brain axis, ageing, sarcopenia, neurodegeneration

## Abstract

This narrative review analyzes how myokines act as key links in communication between skeletal muscle and the brain, focusing on the impact of aging on this axis. Although the current literature links defects in this signaling pathway to sarcopenia, neurodegeneration, and cognitive decline, we still do not know exactly what role each molecule plays individually. Among the main gaps in our understanding are questions about how these substances are transported to the nervous system, what their specific receptors are, and what cause-and-effect relationship actually exists between losing muscle mass and developing neurological problems. In fact, in humans, it remains unclear whether myokines actually reach the brain in truly effective concentrations or whether their benefits are due more to peripheral metabolic, vascular, and inflammatory changes. Today, most of the mechanistic evidence comes from animal and cellular models, while observational studies predominate in humans. This is further complicated by uncertainties regarding the actual origin of these proteins in tissues, their passage through the blood–brain barrier, and their measurement in the blood, making it difficult to determine whether these proteins act as causes, consequences, or simply compensatory responses to aging and disease. On the other hand, although exercise remains the most robust tool for maintaining muscle health and protecting cognitive function, it is still unclear whether we could replicate those same benefits by isolating and stimulating specific myokines. The major challenge for the future is to develop human studies that combine standardized biomarkers with long-term monitoring of muscle function, cognition, and brain structure. Only then will we know whether this fascinating muscle–brain axis can move beyond being a promising hypothesis to become a real therapeutic target.

## 1. Introduction

With ageing, skeletal muscle mass, strength, and physical performance progressively decline, contributing to sarcopenia, frailty, disability, and loss of independence. Sarcopenia is increasingly recognized as a complex systemic disorder, rather than simply loss of muscle. It is characterized by mitochondrial dysfunction, impaired proteostasis, chronic inflammation, metabolic alterations, neuromuscular dysfunction and reduced regenerative capacity [1,2]. Skeletal muscle also functions as an endocrine system organ by releasing myokines and other signaling molecules that participate in local and systemic communication and influence metabolism, inflammatory, and tissue homeostasis [3,4].

The biological effects of muscle ageing may extend beyond the musculoskeletal system. Skeletal muscle and the brain interact via neural, endocrine, metabolic, and inflammatory pathways, supporting the concept of a bidirectional muscle–brain axis [4,5]. Age-related alterations in neuromuscular function, systemic metabolism, inflammation, and muscle secretory activity may therefore affect brain homeostasis, while neurological dysfunction and reduced physical activity can further contribute to muscle deterioration [5,6]. This interaction may partly explain why sarcopenia, cognitive decline, and neurodegenerative disorders often coexist in older adults.

The main physiological mechanisms that interconnect sarcopenic skeletal muscle and cerebral cognitive decline within the framework of cognitive frailty are described below.

Chronic Inflammation (Inflammaging): Elevated levels of pro-inflammatory mediators, such as interleukin-6 (IL-6), tumor necrosis factor alpha (TNF-α), and C-reactive protein (CRP), simultaneously induce muscle protein degradation, leading to atrophy and weakness, and activate microglia in the central nervous system, promoting neurodegeneration and global and hippocampal atrophy [7]. CRP is established as a marker that directly links muscle quality to cognitive performance [8].

It has been suggested that myokines and exercise-responsive signaling molecules are potential mediators of this communication. fibronectin type III domain-containing protein 5 (FNDC5)/irisin is among the best-characterized candidates, with links to hippocampal function, neurogenesis, and cognitive performance. Experimental evidence suggests that peripheral manipulation of irisin can modulate central signaling and phenotypes associated with Alzheimer’s disease [9]. Cathepsin B (CTSB) has also been proposed as a mediator of muscle–brain communication that responds to exercise. Increases in circulating CTSB following aerobic exercise have been associated with cognitive performance in individuals at risk of Alzheimer’s disease [10]. IL-6, fibroblast growth factor 21 (FGF21), insulin-like growth factor 1 (IGF-1), lactate, and other exercise-responsive signals may also be involved in muscle–brain communication, but their biological effects depend largely on tissue source, physiological context, receptor distribution, and duration of exposure [3,4,11].

Therefore, it is unlikely that the link between myokine signaling, sarcopenia, and cognitive decline operates through a single molecular pathway. Rather, ageing may modify the composition and activity of the muscle secretome via reduced physical activity, mitochondrial dysfunction, metabolic disturbances, inflammation, and impaired muscle quality [1,4]. These changes may alter endocrine communication with the brain and affect neuroinflammation, synaptic plasticity, neurogenesis, mitochondrial homeostasis, and protein aggregation [4,6]. However, current evidence is insufficient to establish altered myokine signaling as a direct causal mechanism linking sarcopenia to neurodegeneration. Human studies often show associations, whereas mechanistic studies are mostly based on experimental models.

These limitations highlight several important gaps in our knowledge. The pathways through which individual myokines access the brain are not fully understood, and it remains unclear whether their neurological effects result from direct passage across the blood–brain barrier (BBB), signaling at the BBB, receptor-mediated activity in peripheral tissues, or indirect metabolic and inflammatory pathways [4,9]. Furthermore, many candidate myokines have poorly defined receptor specificity and tissue distribution, and circulating concentrations may not accurately reflect muscle production, molecular processing, or biological activity. Such limitations make it difficult to determine whether altered myokine signaling is a driver, consequence, or adaptive response to ageing and reduced physical activity.

In recent years, science has strongly reinforced the idea that our muscles and brain communicate closely as we age. Recently, Dharmansyah and his team provided a very comprehensive review of how myokines and exercins act as molecular links between sarcopenia and cognitive decline, synthesizing the available data on various candidate molecules and potential prevention strategies. Their work highlights how the loss of muscle function, the disruption of these signals, and mental decline converge, without overlooking the enormous challenges involved in translating these findings into clinical practice. For this reason, in this review we adopt a critical stance focused on making the leap from simple association to proven causality. Rather than merely repeating yet another general overview of myokines in aging, we analyze, step by step, the biological requirements for a molecule to be considered a true messenger between muscle and brain, clearly pointing out which aspects still lack sufficient evidence. The ultimate goal is to lay out which parts of this muscle–brain axis have solid mechanistic support and which require further research before they can be considered reliable biomarkers or therapeutic targets [12].

However, despite the large number of published studies, there are still many unanswered questions about the exact mechanisms by which myokines produced in the muscle influence the brain. To this day, we still do not fully understand their exact cellular origin, how they travel through the bloodstream, how they cross the blood–brain barrier, what their specific receptors are in brain tissue, or the actual direction of cause and effect between sarcopenia and neurological problems.

## 2. The Muscle–Brain Axis: Biological Basis

There is growing recognition that skeletal muscle is an endocrine organ that releases a complex secretome composed of cytokines, growth factors, metabolites, lipids, nucleic acids, and extracellular vesicles to communicate with distant tissues [13]. Myokines are signaling molecules produced by muscle that can act locally through autocrine and paracrine mechanisms or on distant organs through endocrine pathways. Skeletal muscle is an important source of exercise-responsive signals that support adaptations in several organs, including the brain, within the broader framework of exerkines [3,4].

Muscle contraction, exercise type, metabolic state, and training adaptations influence the highly dynamic nature of the skeletal muscle secretome [13,14]. Exercise modifies the expression and secretion of numerous muscle-derived molecules through the activation of intracellular pathways, including AMP-activated protein kinase (AMPK), calcium-dependent signaling pathways, mitogen-activated protein kinases (MAPKs), and the transcriptional coactivator peroxisome proliferator-activated receptor gamma coactivator 1-alpha (PGC-1α) [15]. Thus, muscle-derived communication is not limited to traditional protein myokines but also involves metabolites, microRNAs, lipids, and extracellular vesicles, which participate in inter-organ signaling [3,4].

Contraction-associated increases in intracellular Ca^2+^ are sensed by calmodulin and engage calcium/calmodulin-dependent protein kinase signaling. Calcium/calmodulin-dependent protein kinase II (CaMKII) is particularly relevant in human skeletal muscle and can connect calcium signals to transcriptional regulation involving cAMP response element-binding protein (CREB). Calcium/calmodulin-dependent protein kinase IV (CaMKIV) should not be presented as an equally established mediator in adult human muscle, where its expression has not been consistently detected. Calcium/calmodulin-dependent protein kinase kinase β (CaMKKβ, also termed CaMKK2) can activate AMPK in appropriate cellular contexts; however, its requirement during muscle contraction is disputed. In mouse skeletal muscle, genetic deletion or selective pharmacological inhibition of CaMKK2 did not impair contraction-stimulated AMPK activation or glucose uptake [16].

Exercise-associated redox signals also engage the Kelch-like ECH-associated protein 1 (Keap1)–nuclear factor erythroid 2-related factor 2 (Nrf2) system. Keap1 normally promotes Nrf2 ubiquitination and degradation; redox-dependent changes in this regulation permit Nrf2 accumulation and transcriptional responses through antioxidant response elements. In experimental skeletal muscle, NADPH oxidase 4 (NOX4)-derived hydrogen peroxide supported Nrf2-associated antioxidant responses and mitochondrial adaptations, whereas muscle NOX4 deficiency impaired these responses [17]. These intracellular adaptations provide a physiological context for exercise-responsive muscle signaling, but do not themselves demonstrate an endocrine effect on the brain.

Skeletal muscle can communicate with the brain through several pathways. Some signaling molecules can reach the central nervous system and directly affect neural cells, whereas other circulating muscle-derived substances can interact with receptors at the blood–brain barrier (BBB) [4,18,19]. Muscle-derived metabolites represent another relevant mode of communication. For example, lactate produced during exercise can function both as a metabolic substrate and as a signaling molecule, suggesting that exercise-induced metabolic changes may contribute to brain adaptations [7]. Likewise, extracellular vesicles released by skeletal muscle can transport proteins, lipids, and nucleic acids to distant tissues, providing another pathway for inter-organ communication [3,4].

The effects of muscle-derived signals are not limited to direct actions on neurons. Myokines and myometabolites can modify the brain environment by influencing cellular metabolism, inflammation, and vascular function. Therefore, the muscle–brain axis should be understood as a multifaceted communication network that integrates endocrine, metabolic, vesicular, and indirect systemic mechanisms [3,4].

Muscle–brain communication involves multiple candidate signals, including irisin, derived from fibronectin type III domain-containing protein 5 (FNDC5), interleukin-6 (IL-6), and muscle-derived metabolites such as lactate. Their proposed contributions involve distinct routes and levels of evidence, rather than a single established endocrine pathway [4]. The molecule-specific mechanisms and limitations of the available evidence are discussed in Section 5.

Evidence of functional communication between skeletal muscle and the nervous system also comes from experimental models. For example, muscle-derived neurturin (NRTN), an exercise-induced myokine, has been shown to link skeletal muscle oxidative metabolism with motor neuron properties. Increasing NRTN expression in muscle improved muscle oxidative capacity and promoted a shift toward a slow motor neuron identity in experimental mice [20]. These findings indicate that muscle-derived signals can actively contribute to neuromuscular adaptation [4,20].

Communication between skeletal muscle and the brain is not limited to exercise-induced signals, as cellular stress may also initiate systemic responses. Through an Amyrel/maltose-dependent mechanism in *Drosophila*, Rai et al. demonstrated that proteasome stress in skeletal muscle triggered a long-range adaptive response that delayed age-related changes in proteostasis in the brain and retina [21]. This study provides experimental evidence that skeletal muscle can transmit information about its cellular state to distant neural tissues, although the relevance of this pathway to human physiology remains unknown [21].

The muscle–brain axis is therefore intrinsically bidirectional. While the nervous system regulates muscle physiology through motor innervation, neuromuscular junction activity, and neurotrophic signaling, skeletal muscle can influence neuronal metabolism, plasticity, and neuromuscular function [4,20]. This reciprocal communication may become particularly important during ageing, when alterations in metabolic function, inflammation, and brain homeostasis may coexist with reductions in muscle mass and physical activity.

Ageing may affect the muscle–brain axis through several mechanisms, including the production and secretion of muscle-derived factors, tissue responsiveness, receptor signaling, vascular transport, and BBB function [3,4]. Reduced muscle activity and muscle atrophy may alter the muscle secretome and weaken signaling pathways involved in brain maintenance [4]. However, it remains unclear to what extent age-related changes in specific myokines directly contribute to neurodegeneration or cognitive decline. Further research is particularly needed to clarify cellular targets, myokine transport, receptor specificity, and causal relationships [4]. Addressing these questions will be essential to determine whether alterations in muscle-derived signaling are a consequence of ageing or actively contribute to the coexistence of sarcopenia and neurological dysfunction.

## 3. Ageing and Disruption of the Muscle–Brain Axis

Ageing is a complex, multifactorial biological process that simultaneously affects multiple physiological systems. In this context, the muscle–brain axis has emerged as a dynamic, bidirectional communication system in which skeletal muscle performs not only mechanical functions but also endocrine and metabolic roles. Studies have shown that muscle acts as a secretory organ capable of releasing myokines that may influence brain function, modulating processes such as neuroplasticity, inflammation, and energy metabolism [5].

However, during ageing, this communication may become progressively impaired through the interaction of multiple pathophysiological factors. These factors include loss of muscle mass, increased systemic inflammation, anabolic resistance (a reduced muscle protein-synthetic response to anabolic stimuli), and endocrine alterations, which collectively contribute to dysfunction in the muscle–brain axis. This dysfunction may affect muscle function and has been associated with cognitive decline and an increased risk of neurodegenerative diseases [22,23].

### 3.1. Loss of Muscle Mass and Function

Sarcopenia is one of the most characteristic phenomena of ageing and is characterized by the progressive decline in muscle mass, strength, and physical performance. This process involves an imbalance between the synthesis and degradation of muscle proteins, as well as changes in innervation and muscle tissue quality. Evidence indicates that sarcopenia is not an isolated process but is closely associated with cognitive impairment [24]. Clinically, it has been observed that individuals with lower muscle mass have a higher risk of cognitive decline and dementia, suggesting the existence of shared mechanisms between muscle and brain. These mechanisms may include reduced production of neuroprotective myokines, increased oxidative stress, and mitochondrial dysfunction, all of which may contribute to both muscle and neuronal degeneration [5,25,26,27,28].

### 3.2. Low-Grade Chronic Inflammation

Ageing is associated with persistent low-grade systemic inflammation, termed inflammageing. In older adults, higher circulating IL-6 concentrations have been associated with sarcopenia; this association does not identify skeletal muscle as the source of IL-6 or establish that IL-6 causes cognitive decline [29,30,31]. The distinction between transient exercise-related signaling and sustained inflammation is discussed in Section 5.2.

### 3.3. Anabolic Resistance and Impaired Protein Synthesis

Another key change associated with ageing is anabolic resistance, defined as a reduced skeletal muscle response to anabolic stimuli such as physical exercise or protein intake. This phenomenon is related to alterations in intracellular signaling pathways such as mechanistic target of rapamycin (mTOR) and IGF-1 signaling, which are important regulators of protein synthesis [32].

As a result, ageing muscle exhibits a reduced capacity for regeneration and adaptation, accelerating muscle loss. Furthermore, anabolic resistance can also affect the secretion of exercise-induced myokines, potentially diminishing their positive impact on the brain. This may further impair communication between the two organs [33].

### 3.4. Alterations in Endocrine Signaling

Ageing involves a progressive decline in the production and action of various anabolic hormones, such as testosterone, growth hormone (GH), and insulin-like growth factor 1 (IGF-1) [31]. These hormones play an essential role in maintaining muscle mass and metabolic function. Disruption of this endocrine balance not only contributes to sarcopenia but can also alter brain homeostasis. Hormonal alterations may therefore indirectly affect the muscle–brain axis by modifying the metabolic environment and interorgan signaling. This illustrates how ageing can affect multiple levels of biological regulation [34].

### 3.5. Decrease in Neuroprotective Myokines

Changes in exercise-responsive factors may contribute to altered muscle–brain communication during ageing. However, the effects and tissue sources of individual factors differ, and evidence should not be interpreted as demonstrating a uniform decline in all neuroprotective myokines [29,31]. The molecule-specific evidence and its limitations are discussed in Section 5.

### 3.6. Consequences for the Muscle–Brain Axis

Taken together, interactions among sarcopenia, inflammation, anabolic resistance, endocrine alterations, and changes in myokine secretion may contribute to disruption of the muscle–brain axis. This disruption may help explain an association between physical and cognitive decline among older adults [34,35,36].

## 4. Molecular Mechanisms Linking Sarcopenia and Neurodegeneration

The pathophysiological interconnection between sarcopenia and neurodegeneration during ageing may involve bidirectional communication along the muscle–brain axis [37,38,39]. Skeletal muscle acts as an endocrine organ, and selected muscle-related factors have shown effects on neural phenotypes in experimental models. These findings support biological plausibility, but do not establish that altered myokine secretion causes the association between sarcopenia and neurodegeneration in humans [25,28,40,41]. The evidence for individual factors is evaluated in Section 5.

**Figure 1:**
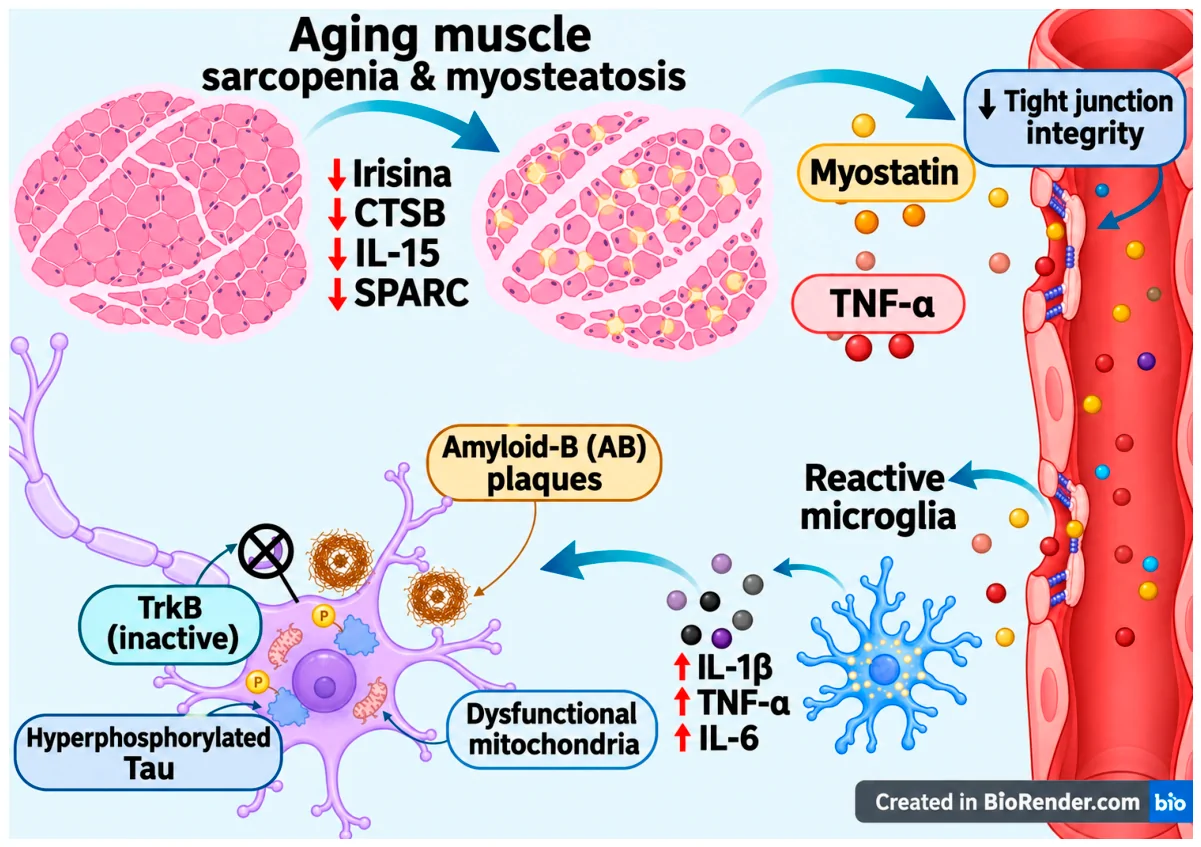
Age-related disruption of the muscle–brain axis and its potential contribution to neurodegeneration. The diagram integrates proposed relationships among sarcopenia, myosteatosis, altered muscle-related signaling, blood–brain barrier dysfunction, glial activation, and neuronal pathology. The arrows represent a conceptual model rather than a demonstrated causal sequence in humans. The depicted changes in individual factors should not be interpreted as uniform findings across all populations or disease stages.

Age-related changes may involve altered muscle-derived signaling, vascular dysfunction, and neuroinflammatory responses. Figure 1 presents a conceptual summary of these proposed relationships; the individual pathways are discussed below.

### 4.1. Chronic Inflammation

Biological ageing is commonly associated with a state of systemic, sterile, chronic, and low-grade inflammation known as inflammageing. This persistent inflammatory state may contribute to anabolic resistance in muscle and neurotoxicity in the central nervous system (CNS) [8,29,37,42].

#### 4.1.1. Interleukin-6 (IL-6)

A cytokine with a dual role in the muscle–brain axis. During exercise, muscle releases IL-6 as a myokine, promoting glucose uptake and fatty acid oxidation through AMPK. In contrast, chronically elevated IL-6 with ageing and sedentary behavior is associated with sarcopenia and muscle weakness [8,42].

#### 4.1.2. Tumor Necrosis Factor Alpha (TNF-α)

TNF-α is a key catabolic cytokine whose serum and intramuscular levels may increase during ageing. In skeletal muscle, TNF-α disrupts the insulin receptor signaling cascade by inducing inhibitory phosphorylation at serine residues of insulin receptor substrate 1 (IRS-1), thereby promoting insulin resistance and disuse muscle atrophy [30,38,42].

Similarly, TNF-α may reduce the expression and stability of critical myogenic transcription factors such as MyoD, thereby impairing the activation and fusion of satellite cells necessary for muscle regeneration and maintenance. In the CNS, TNF-α may promote death receptor-mediated neuronal apoptosis and alters the function of glial glutamate transporters, contributing to synaptic excitotoxicity and accelerating neurodegenerative processes in diseases such as Alzheimer’s disease and Parkinson’s disease [8,37,42,43].

#### 4.1.3. Nuclear Factor Kappa B (NF-κB)

NF-κB is the central transcriptional signaling node where cellular inflammatory and catabolic signals converge. Binding of TNF-α to its membrane receptor can activate IκB kinase (IKK), which phosphorylates the inhibitor IκB, allowing the NF-κB dimer to translocate freely into the cell nucleus [37,43]. In contrast, IL-6 signals primarily through the Janus kinase (JAK)/signal transducer and activator of transcription 3 (STAT3) pathway, although crosstalk with NF-κB signaling may occur.

In skeletal muscle, inflammatory pathways involving NF-κB can contribute to increased protein catabolism. Chronic inflammation is also implicated in cognitive impairment, providing a possible shared pathological context for muscle and brain dysfunction. These observations should not be interpreted as demonstrating that muscle inflammation causes irreversible neuronal damage or synaptic loss in humans [42,44].

### 4.2. Oxidative Stress

Redox imbalance, characterized in part by increased production of reactive oxygen species (ROS), can impair both skeletal muscle and neural tissue [37,43,45].

#### 4.2.1. Reactive Oxygen Species (ROS)

During ageing, redox imbalance may impair skeletal muscle and neural tissue. Mitochondrial superoxide and hydrogen peroxide can contribute to oxidative modification of proteins, lipids, and DNA. Superoxide can also participate in the formation of peroxynitrite, linking oxidative and nitrative stress [40].

Antioxidant defenses should be considered as interacting systems rather than as uniformly depleted enzymes. In a *Sod1*-deficient mouse model of accelerated sarcopenia, muscle-specific peroxiredoxin 3 overexpression improved mitochondrial and contractile function, while neuromuscular junction impairment persisted. This supports a compartment-specific protective effect, not proof of reversal of human sarcopenia [40].

Thioredoxins recycle oxidized peroxiredoxins; glutaredoxins regulate protein thiol modifications, and glutathione reductase regenerates reduced glutathione. Heme oxygenase 1 participates in heme catabolism and stress responses. These systems can undergo adaptive or damaging changes; the reviewed evidence does not justify assuming that all decline uniformly in human sarcopenia. Peroxynitrite-derived species can nitrate tyrosine residues, adding nitrative damage to protein oxidation and lipid peroxidation [41].

#### 4.2.2. Mitochondrial Damage

Chronic oxidative stress can damage mitochondrial DNA (mtDNA) in skeletal muscle and neural cells. Mitochondrial DNA is particularly susceptible to oxidative damage because of its proximity to the electron transport chain and differences in its protective and repair mechanisms compared with nuclear DNA. The accumulation of mtDNA mutations and deletions can impair the synthesis of essential electron transport chain subunits, reducing ATP production and cellular bioenergetic capacity [37,43].

Damaged mitochondria may undergo membrane depolarization and opening of the mitochondrial permeability transition pore (mPTP). The subsequent release of cytochrome c into the cytosol activates the caspase cascade and may contribute to myocyte atrophy and neuronal death. Mitochondrial damage can also release damage-associated molecular patterns (DAMPs), including oxidized mtDNA and mitochondrial N-formyl peptides. These molecules activate innate immune pathways, including Toll-like receptor 9 (TLR9) and the NLR family pyrin domain containing 3 (NLRP3) inflammasome, thereby amplifying sterile inflammation in skeletal muscle and neural tissue [37,43,46].

### 4.3. Mitochondrial Dysfunction

#### 4.3.1. Peroxisome Proliferator-Activated Receptor Gamma Coactivator 1-Alpha (PGC-1α)

PGC-1α is a central transcriptional coactivator involved in mitochondrial biogenesis, cellular respiration, and antioxidant defense. Through interactions with nuclear respiratory factors and mitochondrial transcription factor A (TFAM), PGC-1α regulates the expression of nuclear-encoded mitochondrial genes and supports mtDNA replication and transcription. In skeletal muscle, PGC-1α also regulates the expression of potentially neuroprotective myokines, including irisin through its precursor FNDC5, and fibroblast growth factor 21 (FGF21) [27,46,47,48].

Physical inactivity and ageing may reduce PGC-1α expression in skeletal muscle, contributing to lower mitochondrial density and impaired oxidative phosphorylation. Reduced PGC-1α activity may also decrease FNDC5/irisin signaling, potentially weakening peripheral signals involved in hippocampal brain-derived neurotrophic factor (BDNF) expression and cognitive function [27,46,47].

#### 4.3.2. AMP-Activated Protein Kinase (AMPK)

AMPK is a key cellular energy sensor that helps restore ATP homeostasis during metabolic stress. In skeletal muscle, contraction-associated changes in the AMP-to-ATP ratio activate AMPK, which can stimulate mitochondrial biogenesis partly through the phosphorylation and activation of PGC-1α [46].

Ageing and ectopic lipid accumulation in skeletal muscle may attenuate AMPK signaling and reduce the adaptive activation of PGC-1α. Impaired AMPK signaling may also compromise mitophagy, leading to the accumulation of dysfunctional mitochondria and reduced energy production in muscle and neural tissue [38,46].

#### 4.3.3. Sirtuin 1 (SIRT1)

SIRT1 is a nicotinamide adenine dinucleotide (NAD+)-dependent deacetylase that interacts with AMPK in the regulation of cellular metabolism. SIRT1 deacetylates PGC-1α, thereby enhancing its transcriptional activity. In turn, AMPK can promote NAD+ availability by regulating nicotinamide phosphoribosyltransferase (NAMPT), supporting SIRT1 activity. Age-related reductions in tissue NAD+ levels may therefore decrease SIRT1 activity and impair mitochondrial homeostasis [38,46].

Altered SIRT1 signaling may affect mitochondrial homeostasis and stress responses. However, a single obligatory AMPK–SIRT1–transcription factor EB (TFEB) pathway linking muscle dysfunction to amyloid-β and tau accumulation in the human brain has not been established by the evidence presented here.

#### 4.3.4. PGC-1α and Nrf2-Associated Antioxidant Responses

Experimental evidence links PGC-1α to nuclear factor erythroid 2-related factor 2 (Nrf2) in skeletal muscle redox regulation. In 7β-hydroxycholesterol-treated C2C12 cells, PGC-1α overexpression increased Nrf2 expression and reduced cellular and mitochondrial ROS signals, whereas PGC-1α silencing produced the opposite effects. In aged mice, muscle-specific PGC-1α deficiency was associated with lower gastrocnemius Nrf2 expression and poorer muscle function. Treatment with diethylmaleate, used as an Nrf2 activator, improved grip strength and hanging performance and increased muscle Nrf2, heme oxygenase-1, and NAD(P)H quinone oxidoreductase 1 protein levels. These findings support a functional relationship between PGC-1α and Nrf2-associated antioxidant responses in experimental muscle models, but do not establish direct transcriptional regulation, clinical efficacy, or a myokine-mediated effect on the brain [49].

### 4.4. PGC-1α-Associated Substrate Metabolism and Muscle Protein Synthesis

PGC-1α-associated metabolic remodeling involves distinct transcriptional partners. In high-fat diet-fed mice, pharmacological activation of PPARβ/δ restored skeletal muscle PGC-1α and PPARα protein levels and increased the expression of fatty acid oxidation-related genes [50]. For amino acid metabolism, silencing estrogen-related receptor α (ERRα) abolished PGC-1α-induced upregulation of branched-chain amino acid catabolic genes in primary human myotubes, identifying ERRα as a necessary partner for this transcriptional response [51].

Amino acid uptake and protein synthesis require separate consideration. In mice, resistance running increased soleus leucine uptake and enhanced leucine-stimulated mechanistic target of rapamycin complex 1 (mTORC1) signaling and muscle protein synthesis for up to 48 h, without establishing dependence on PGC-1α or PPARα [52]. In a separate mouse study of intermittent prednisone treatment, myocyte PGC-1α was required for treatment-induced increases in muscle protein synthesis, whereas isoform-specific rescue experiments distinguished the contribution of PGC-1α1 to mitochondrial abundance from that of PGC-1α4 to muscle mass [53]. These studies do not establish PGC-1α-dependent PPARα activation as a common mechanism for fatty acid uptake, amino acid uptake, and selective contractile protein synthesis.

### 4.5. PGC-1α and MEF2-Associated Muscle Fiber Remodeling

PGC-1α-dependent metabolic remodeling can accompany changes in muscle fiber identity. In transgenic mice, PGC-1α overexpression reduced the proportion of type IIB fibers and increased type I and IIA fibers. In *Myh4*-null mice, it also shifted fiber composition toward type IIA and partially preserved muscle mass and fiber number during postnatal growth. These findings support an experimental link between PGC-1α and oxidative fiber remodeling [54].

The functional state of PGC-1α transcriptional partners, including myocyte enhancer factor 2 (MEF2), also merits consideration. In a mouse model of spinal and bulbar muscular atrophy, sequestration of MEF2 and other transcriptional regulators in intranuclear inclusions accompanied altered PGC-1α target-gene regulation and myonuclear identity, despite unchanged PGC-1α abundance. These observations support a context-dependent relationship between PGC-1α signaling and MEF2 function, rather than universal induction of MEF2 by PGC-1α [55]. Neither model establishes that this mechanism prevents age-related fiber loss in humans.

## 5. Myokines Involved in Muscle–Brain Communication

Skeletal muscle is recognized as an endocrine organ capable of releasing a wide range of bioactive molecules, collectively known as myokines, that act through autocrine, paracrine, and endocrine mechanisms. These signals contribute to communication between skeletal muscle and the brain and may influence energy metabolism, neuronal plasticity, cell survival, and inflammatory responses, thereby linking muscle function to central nervous system homeostasis [38].

### 5.1. Neuroprotective Myokines

Several exercise-responsive myokines have been implicated in muscle–brain communication through their effects on neurogenesis, synaptic plasticity, neuronal survival, and BDNF regulation. Selected myokines with proposed neuroprotective effects and their mechanisms of action are summarized in Table 1.

The table summarizes the main mechanisms of action, effects on the brain, and potential neuroprotective benefits of selected myokines. Abbreviations: αV/β5, integrin alpha V/beta 5; Akt, protein kinase B; BDNF, brain-derived neurotrophic factor; DCX, doublecortin; ERK, extracellular signal-regulated kinase; gp130, glycoprotein 130; LIF, leukemia inhibitory factor; LIFRβ, leukemia inhibitory factor receptor beta; MAPK, mitogen-activated protein kinase; PI3K, phosphoinositide 3-kinase; TrkB, tropomyosin receptor kinase B. The listed effects are context-dependent and derive largely from experimental models; they do not demonstrate that each factor has a muscle-derived endocrine action in humans.

#### 5.1.1. Irisin

Irisin is generated through the proteolytic cleavage of the transmembrane protein FNDC5, whose expression in skeletal muscle is induced by aerobic exercise through the transcriptional coactivator PGC-1α. Irisin has been reported to signal through αV-containing integrins, including αVβ5, expressed by several cell types in the brain [56]. Peripheral irisin may access the brain or influence it through signaling at the blood–brain barrier, although the mechanisms involved remain incompletely understood. Irisin activates intracellular pathways that include cAMP/PKA/CREB, STAT3, AMPK, and ERK and may increase brain-derived neurotrophic factor (BDNF) expression. These effects have been associated with enhanced synaptic plasticity, hippocampal neurogenesis, and memory. In mouse models of Alzheimer’s disease, reduced FNDC5/irisin signaling exacerbates cognitive deficits and amyloid pathology, whereas peripheral administration of irisin has been reported to improve memory function [57]. In clinical studies, cerebrospinal fluid irisin levels have been reported to be lower in patients with Alzheimer’s disease and positively associated with cognitive status. However, its clinical use as a biomarker remains limited by concerns regarding the specificity and reliability of currently available commercial immunoassays [56]. Clinical biomarker interpretation remains limited by uncertainty about tissue origin and assay specificity, as discussed in Section 8.3.

#### 5.1.2. Cathepsin B (CTSB)

Cathepsin B is a cysteine protease released by skeletal muscle in response to prolonged aerobic exercise. Although a specific receptor mediating its effects in the brain has not been identified, experimental evidence suggests that circulating CTSB can cross the blood–brain barrier and influence hippocampal cells [58]. Its proposed mechanisms include increasing the expression of BDNF and doublecortin (DCX), thereby supporting adult neurogenesis, as well as modulating amyloid-β metabolism [48]. Experimental studies have shown that CTSB-deficient mice do not exhibit the improvements in spatial memory and hippocampal neurogenesis normally associated with exercise [34]. In humans, exercise-induced increases in circulating CTSB have been associated with improved episodic memory. However, the effects of CTSB appear to be context-dependent, as sustained elevation in inflammatory conditions may contribute to oxidative stress and detrimental microglial activation [38].

#### 5.1.3. BDNF (Brain-Derived Neurotrophic Factor)

Brain-derived neurotrophic factor (BDNF) is a neurotrophin that plays an important role in neuronal survival, synaptic plasticity, neurogenesis, learning, and memory. Unlike myokines, whose endocrine activity depends primarily on their release from skeletal muscle into the circulation, BDNF is produced predominantly in the central nervous system and other tissues. Skeletal muscle can also express BDNF in response to contraction and exercise. Muscle-derived BDNF appears to act mainly through local autocrine and paracrine mechanisms involved in muscle metabolism and regeneration, whereas exercise may indirectly promote BDNF signaling in the brain through muscle–brain communication. This characteristic distinguishes BDNF from myokines with a more clearly established peripheral endocrine function [33,34,56,59].

In the brain, BDNF binds to the tropomyosin receptor kinase B (TrkB) and activates intracellular pathways such as PI3K/Akt and MAPK/ERK, promoting neuronal survival, dendritic spine formation, and synaptic plasticity. These mechanisms contribute to the beneficial effects of exercise on hippocampal function, learning, and memory. Experimental studies have shown that inhibition of BDNF/TrkB signaling attenuates the neuroplastic and cognitive benefits associated with exercise. In humans, aerobic exercise has been associated with changes in circulating BDNF concentrations and markers of hippocampal structure and function. However, peripheral BDNF levels should be interpreted cautiously because a substantial proportion of blood BDNF is stored in and released from platelets, making its tissue of origin difficult to determine [33,34,56,59].

#### 5.1.4. LIF (Leukemia Inhibitory Factor)

Leukemia inhibitory factor (LIF), a member of the IL-6 cytokine family, is expressed in skeletal muscle in response to contraction, particularly during resistance exercise. LIF signals through a receptor complex composed of LIFRβ and gp130 and activates intracellular pathways involved in cell proliferation and differentiation. In neural tissue, LIF has been implicated in neural progenitor cell regulation and protection against amyloid-β-induced toxicity. Experimental studies have reported that LIF administration reduces apoptosis in hippocampal neuronal cultures exposed to amyloid-β peptides. Changes in circulating LIF following acute exercise have also been observed in individuals with mild cognitive impairment, although their physiological significance remains uncertain. Importantly, exercise-induced LIF may act primarily within skeletal muscle through autocrine or paracrine mechanisms, and the contribution of muscle-derived LIF to brain function remains unclear [38,60,61].

### 5.2. Inflammatory and Immunomodulatory Myokines

#### 5.2.1. IL-6 (Interleukin 6)

IL-6 should not be classified as uniformly anti-inflammatory, because its biological effects depend on the physiological and inflammatory context. Its release from skeletal muscle during physical activity should be distinguished from its involvement in chronic inflammation [11]. A systematic review and meta-analysis of cross-sectional studies found higher serum IL-6 concentrations in older adults with sarcopenia than in those without sarcopenia. However, these observational findings do not establish causality or identify skeletal muscle as the source of circulating IL-6 [30].

#### 5.2.2. IL-10 (Interleukin 10)

Although IL-10 is produced primarily by immune cells, exercise may increase its circulating concentration and expression in skeletal muscle. IL-10 acts through the IL-10 receptor complex to suppress macrophage activation and reduce the production of pro-inflammatory cytokines such as TNF-α and IL-6. These effects may attenuate microglia-mediated neuroinflammation and protect neuronal integrity. Experimental studies in IL-10-deficient mice have reported accelerated age-related muscle loss and increased systemic inflammation. Exercise interventions have also been associated with increased circulating IL-10, although evidence demonstrating corresponding changes in the brains of patients with Alzheimer’s disease remains limited. In severe sarcopenia, elevated IL-10 levels may reflect a compensatory response to persistent systemic inflammation rather than an effective anti-inflammatory response [29,59].

#### 5.2.3. IL-15 (Interleukin 15)

IL-15 is expressed in skeletal muscle, and its production may be influenced by resistance exercise. It signals through a receptor system involving IL-15Rα, IL-2/15Rβ, and the common γ-chain. IL-15 commonly binds with high affinity to IL-15Rα and is presented to neighboring cells expressing the βγ receptor complex. Its proposed effects include reducing TNF-α-mediated muscle apoptosis and improving insulin sensitivity. In the brain, the effects of IL-15 appear to be context-dependent: physiological signaling may help regulate inflammatory responses, whereas excessive or prolonged signaling may promote microglial activation. Experimental overexpression of IL-15 in mice has been associated with reduced visceral adiposity and preservation of lean mass, while lower circulating IL-15 levels have been reported in older adults with sarcopenia. Its therapeutic application is limited by its short circulating half-life and dependence on receptor availability and mode of presentation [38,62,63].

#### 5.2.4. SPARC (Osteonectin)

Secreted protein acidic and rich in cysteine (SPARC), also known as osteonectin, is a matricellular protein released by skeletal muscle during exercise and tissue regeneration. SPARC contributes to extracellular matrix organization, cytoskeletal remodeling, and muscle repair. Experimental evidence suggests that SPARC may also influence amyloid-β metabolism; however, evidence directly linking muscle-derived circulating SPARC to amyloid-β clearance in the human brain remains limited. Circulating SPARC levels may increase after acute exercise in humans, but its role in muscle–brain communication and its clinical relevance in neurodegenerative disease require further investigation [25,38,60,61].

### 5.3. Metabolic Myokines

#### 5.3.1. FGF21 (Fibroblast Growth Factor 21)

FGF21 is produced primarily by the liver, although skeletal muscle and adipose tissue can also produce it under conditions such as mitochondrial stress and exercise. FGF21 signals through fibroblast growth factor receptors, particularly FGFR1c, in association with the β-Klotho coreceptor. Evidence indicates that circulating FGF21 can access the central nervous system and influence neuronal metabolism and cerebrovascular function. In experimental models, FGF21 reduces oxidative stress, supports mitochondrial function, and protects against neuronal apoptosis. It has also been reported to attenuate cerebrovascular injury in models of type 2 diabetes. In humans, exercise-induced changes in circulating FGF21 have been associated with systemic metabolic adaptations, although responses vary according to exercise intensity, baseline metabolic status, and FGF21 sensitivity [5,33,38,48,61].

#### 5.3.2. BAIBA (β-Aminoisobutyric Acid)

β-Aminoisobutyric acid (BAIBA) is a nonprotein amino acid released by skeletal muscle in response to exercise. It influences metabolic pathways that include AMPK and PI3K/Akt signaling and has been associated with increased fatty acid oxidation and the browning of white adipose tissue. Experimental studies suggest that BAIBA may protect neural cells against oxidative stress and support cellular energy homeostasis. BAIBA administration has also improved insulin sensitivity and reduced adiposity in animal models. In humans, circulating BAIBA has been associated with physical activity and metabolic health, but its validity as a biomarker of physical fitness has not been established. Moreover, its ability to cross the human blood–brain barrier and the contribution of muscle-derived BAIBA to brain function remain incompletely characterized [58,60].

#### 5.3.3. Apelin

Apelin is an exercise-responsive peptide produced by several tissues, including skeletal muscle and adipose tissue. It is an endogenous ligand of the G protein-coupled apelin receptor, also known as APJ or APLNR. Apelin activates intracellular pathways that include PI3K/Akt/mTOR and has been implicated in cell survival, vascular regulation, and tissue regeneration. Its proposed effects in the brain include promoting angiogenesis, improving cerebral microcirculation, and reducing neuroinflammation. In streptozotocin-induced rat models of cognitive impairment, apelin administration has been reported to improve memory and reduce microglial activation. Acute aerobic exercise may also alter circulating apelin levels in humans, although the response appears to vary according to exercise and participant characteristics. A major limitation to its therapeutic application is its short half-life in the systemic circulation [33,58,61,64].

These myokines may contribute to muscle–brain communication by modulating inflammatory responses, energy metabolism, oxidative stress, and neurovascular function. The main anti-inflammatory and metabolic myokines analyzed in this review are summarized in Table 2.

The table summarizes the main anti-inflammatory, metabolic, and neurovascular myokines involved in muscle–brain crosstalk and their potential protective effects. Abbreviations: Akt, protein kinase B; AMPK, AMP-activated protein kinase; APJ, apelin receptor; BAIBA, β-aminoisobutyric acid; FGF21, fibroblast growth factor 21; IL-6, interleukin-6; IL-10, interleukin-10; IL-15, interleukin-15; mTOR, mechanistic target of rapamycin; PI3K, phosphoinositide 3-kinase; SPARC, secreted protein acidic and rich in cysteine; TNF-α, tumor necrosis factor alpha. Functional categories are descriptive rather than mutually exclusive. The listed effects do not establish clinical efficacy or direct transport from muscle to the human brain.

## 6. Sarcopenia and Neurodegenerative Diseases

The muscle–brain axis is a bidirectional communication system involved in maintaining physiological homeostasis during ageing [58]. Dysfunction of this axis, including altered myokine secretion and impaired muscle function, may contribute to the association between sarcopenia, cognitive decline, and neurodegenerative processes [8,65,66]. This section examines the evidence linking sarcopenia with major neurodegenerative diseases.

### 6.1. Alzheimer’s Disease

Alzheimer’s disease (AD) and sarcopenia frequently coexist in older adults [67,68]. The pooled prevalence of sarcopenia among patients diagnosed with AD is estimated at 33.9% (95% CI: 27.6–40.2%) [68,69], a rate significantly higher than that observed in the general elderly population. This association appears to vary according to the severity of cognitive impairment: the prevalence of sarcopenia is 31.2% (95% CI: 22.3–40.2%) among patients with mild AD and increases to 41.9% (95% CI: 32.1–51.6%) among those with moderate AD [68]. A multivariable logistic regression analysis found that patients with AD had a significantly higher risk of sarcopenia, with an odds ratio (OR) of 2.670 (95% CI: 1.566–4.555) [68]. Specific clinical studies also confirm this independent association, reporting that AD is significantly associated with sarcopenia with an OR of 2.048 (95% CI: 1.049–3.998; *p* = 0.036) [67].

This association is based on a bidirectional and synergistic pathophysiology:

Cognitive and functional decline: Cognitive impairment in Alzheimer’s disease leads to physical inactivity, reduced appetite, malnutrition, and difficulty performing activities of daily living, directly accelerating the loss of muscle mass and strength [70,71].

Altered muscle signaling: Reduced muscle mass alters the secretion of myokines involved in the muscle–brain axis, including irisin, cathepsin B, oncostatin M, BDNF, IGF-1, and LIF. These factors normally promote angiogenesis, neurogenesis, synaptic plasticity, and neuronal metabolism [66].

Furthermore, sarcopenia in Alzheimer’s disease is associated with unfavorable neuroimaging findings, particularly hippocampal atrophy, as well as poorer overall health outcomes and more severe cognitive deficits [66]. Both conditions share common pathogenic mechanisms, including chronic inflammation (inflammageing), mitochondrial dysfunction, increased oxidative stress, and apoptosis [8,65,68].

### 6.2. Parkinson’s Disease

Parkinson’s disease (PD) is the second most common neurodegenerative disease worldwide [72]. Patients with PD have a markedly increased risk of sarcopenia. Prevalence studies indicate that confirmed sarcopenia affects 10.9–31.4% of this population, whereas probable sarcopenia, defined by reduced muscle strength or dynapenia, occurs in 23.9–66.7% of cases [73]. Specifically, the prevalence of low grip strength ranges from 26.2% to 76.7% among patients with PD and related disorders [73]. In a multivariable analysis, PD was independently associated with sarcopenia, with an odds ratio (OR) of 2.744 (95% CI: 1.231–6.116; *p* = 0.014) [67].

Sarcopenia appears to play a significant role in the progression of Parkinson’s disease (PD), contributing to a decline in quality of life and increasing the risk of falls, hip fractures, dysphagia, and overall mortality [72,73]. Although the underlying mechanisms are not fully understood, several processes may contribute to this association. These include the accumulation of α-synuclein in skeletal muscle, the progressive loss of motor neurons and functional motor units, and chronic systemic neuroinflammation characterized, among other factors, by elevated concentrations of interleukin-6 (IL-6), which are associated with muscle atrophy and impaired physical function [72,73,74].

Additionally, kinematic analyses using digital technology demonstrate that sarcopenia profoundly alters the gait patterns characteristic of Parkinson’s disease. The gait parameter showing the strongest negative correlation with sarcopenia is the average range of motion (ROM) of the hip joint. Lower hip ROM is associated with a greater likelihood of sarcopenia, shorter step length, and a predisposition to recurrent falls [74,75]. Sarcopenia has also been found to alter biomechanical coupling between the knee and ankle during gait [74].

### 6.3. Amyotrophic Lateral Sclerosis (ALS) and Neuromuscular Disorders

Amyotrophic lateral sclerosis (ALS) involves progressive motor neuron degeneration and denervation-associated muscle wasting. Although its pathogenesis differs from that of age-related sarcopenia, both conditions involve motor unit loss and muscle atrophy, making ALS a relevant setting for examining interactions between neural degeneration and skeletal muscle dysfunction [76].

Within the scope of this review, these features raise the question of whether altered muscle-derived signaling contributes to neural disease progression in addition to the effects of denervation on muscle. However, muscle wasting alone does not demonstrate a myokine-mediated effect on the brain or spinal cord. Establishing such a contribution would require muscle-specific manipulation of a candidate signal and assessment of neural outcomes, while distinguishing endocrine effects from local neuromuscular interactions. ALS is therefore considered here as a context for investigating this question, rather than as established evidence of myokine-mediated neuroprotection [77].

### 6.4. Huntington’s Disease

Huntington’s disease (HD) is an inherited disorder characterized by expansion of CAG trinucleotide repeats (>35 repeats) in the huntingtin (*HTT*) gene [78]. Although its primary manifestations are neurological and affect the basal ganglia and cerebral cortex, mutant HTT is expressed ubiquitously. Consequently, patients with HD experience substantial peripheral dysfunction, with muscle wasting and progressive weight loss representing two major clinical features [76]. Patients with HD show an average reduction of 50% in isometric muscle strength compared with healthy controls [76].

Muscle pathology in Huntington’s disease is characterized by profound and multifactorial molecular alterations [76].

Mitochondrial dysfunction and metabolic adaptations: Muscle metabolism is markedly altered, with substantial reductions in resting ATP, phosphocreatine, and inorganic phosphate levels in both symptomatic and presymptomatic patients. During post-exercise recovery, the peak mitochondrial ATP production rate is reduced by 44% in symptomatic patients. This reduction is accompanied by a lower anaerobic threshold and increased lactate production, indicating compensatory reliance on glycolysis.

Transcriptional dysregulation and fibrillar atrophy: Quadriceps muscle exhibits a gene dysregulation profile similar to that observed in the brain. As muscle fiber atrophy progresses, fast-twitch type II fibers transition to slow-twitch type I fibers.

Neuromuscular junction dysfunction and excitability: Skeletal muscles in HD models develop hyperexcitability because of reduced expression of muscle chloride channels (ClC-1) and potassium channels (Kir2.1). Altered synaptic transmission at the neuromuscular junction and increased sensitivity to acetylcholine are also observed [79].

Apoptosis, autophagy and mTOR: Muscle wasting involves overactivation of the mTOR signaling pathway, with a 19% increase in overall protein synthesis during atrophic phases. It is also associated with elevated levels of key apoptotic caspases, including caspases 3, 7, 8, and 9, and increased activity of the p53/caspase-6 pathway, which contributes to the degradation of structural proteins.

Role of PGC-1α: PGC-1α (PPARγ coactivator 1α) and its mitochondrial biogenesis-related target genes are markedly reduced in the skeletal muscle of patients with Huntington’s disease [76]. Preclinical studies demonstrate that pharmacologically increasing PGC-1α expression or reducing cytoplasmic mutant huntingtin through manipulation of histone deacetylase 4 (HDAC4) restores mitochondrial morphology, prevents muscle fiber conversion, and reduces mutant huntingtin aggregation, thereby improving motor coordination and extending lifespan. These muscle abnormalities are relevant to the muscle–brain axis because they may change the physiological context of interorgan signaling. Nevertheless, parallel muscle and brain pathology in Huntington’s disease does not demonstrate an endocrine mechanism: mutant huntingtin is expressed in both tissues. Establishing a myokine-mediated contribution would require muscle-specific manipulation and measurement of neural outcomes beyond motor performance [76].

### 6.5. The Interplay Between Sarcopenia and Cognitive Frailty

Cognitive frailty is clinically defined as a geriatric syndrome characterized by the coexistence of physical frailty, weight loss, exhaustion, slow gait, weakness, and low physical activity, and mild cognitive impairment (MCI) in the absence of diagnosed dementia [8,80]. Sarcopenia is now recognized as an important risk factor underlying the bidirectional and synergistic relationship between these two dimensions [8,65,80]. Performance in overall cognitive ability and specific domains, particularly verbal fluency, declines progressively as the severity of sarcopenia and patient immobility increases [8].

Hypothalamic–Pituitary–Adrenal (HPA) Axis Dysfunction: With age, basal cortisol levels increase, and cortisol secretion loses its normal diurnal pattern, promoting muscle catabolism and inducing structural damage in the hippocampus [8]. Conversely, declining levels of androgenic hormones, such as testosterone and dehydroepiandrosterone sulfate (DHEA-S), reduce muscle protein synthesis, resulting in decreased grip strength, and diminish neuroprotective support for synaptic plasticity and β-amyloid clearance [8].

Dysregulation of Energy and Endocrine Homeostasis: Concentrations of insulin-like growth factor 1 (IGF-1) and growth hormone (GH), which synergistically influence extensor muscle strength and gait speed, are markedly reduced in frail individuals. This hormonal dysfunction enhances cytokine-induced myocyte catabolism and exacerbates cerebral and systemic insulin resistance. The accumulation of intramuscular adipose tissue in sarcopenic obesity and changes in appetite-regulating hormones, such as ghrelin and leptin, also act as important metabolic modulators within this axis [8].

Oxidative Stress and Mitochondrial Dysfunction: Loss of mitochondrial redox homeostasis and the accumulation of DNA damage in myocytes lead to sarcomere loss. Excess reactive oxygen species (ROS) impair both the cerebral vascular endothelium and muscle function, accelerating functional decline in both systems [8].

Genomic and Metabolomic Biomarkers: Shared genetic and circulating biomarkers have been associated with both physical and cognitive impairment. These include the *APOE ε4* allele, polymorphisms in genes encoding inflammatory cytokines, and elevated serum neurofilament light chain (sNfL] levels [8,80]. Furthermore, reduced plasma concentrations of certain lysophosphatidylcholine species, particularly LPC 18:2 and LPC 18:1, are associated with systemic inflammation, the development of Alzheimer’s disease, and an accelerated longitudinal decline in gait speed and physical independence among older adults [8].

Within this framework, altered myokine signaling is a candidate mediator rather than a defining feature of cognitive frailty. Future studies should test whether specific muscle-derived signals explain longitudinal changes in cognition after accounting for physical activity, nutritional status, comorbidity, and vascular risk.

## 7. Therapeutic Perspectives

Growing evidence supporting the muscle–brain axis has contributed to a shift in the clinical approach to ageing, from a traditional focus on isolated organ pathologies toward an integrated understanding of pathological crosstalk. Conditions such as sarcopenia, osteoporosis, and Alzheimer’s disease are increasingly recognized as interconnected through complex endocrine and immunometabolic networks that form the muscle–bone–brain axis. Progressive loss of muscle mass and ectopic fat infiltration into skeletal muscle, known as myosteatosis, not only impair motor function but also alter the muscle secretory profile. This alteration is characterized by reduced levels of neuroprotective factors and increased production of pro-inflammatory mediators that may contribute to cognitive decline. Consequently, targeting this axis through multidimensional therapeutic strategies may support coordinated interventions that extend beyond exercise to include pharmacological approaches, gene therapy, extracellular vesicle bioengineering, and precision medicine informed by multi-omics technologies [36,38,47,81].

### 7.1. Exercise

Structured moderate-intensity aerobic exercise, performed three to five times per week for at least 30 min per session, is an important physiological stimulus for brain plasticity and mitochondrial biogenesis through activation of the PGC-1α/FNDC5 pathway. This activation promotes the cleavage of the transmembrane protein FNDC5 and the release of irisin into the circulation. The proposed neural effects involve context-dependent pathways, as discussed in Section 5.1; they should not be represented as one uniformly activated signaling cascade or as a mechanism established in humans [32,34,36,38,57,82].

Resistance training activates anabolic pathways that help counteract sarcopenia, particularly the mammalian target of rapamycin complex 1 (mTORC1) and PI3K/Akt signaling pathways. Muscle contraction during resistance exercise also stimulates the release of anabolic and metabolic factors such as insulin-like growth factor 1 (IGF-1), fibroblast growth factor 21 (FGF21), and osteonectin (SPARC). These factors may improve peripheral insulin sensitivity, promote the regeneration of type II muscle fibers, and support protein-clearance processes involved in limiting amyloid accumulation. Clinically, gains in muscle strength and mass following moderate- to high-intensity resistance training at 70–85% of one-repetition maximum have been associated with improvements in working memory and processing speed. These improvements have also been associated with increased cerebral blood flow to the temporal cortex and preservation of white matter integrity in older adults with mild cognitive impairment [36,38,64].

**Figure 2:**
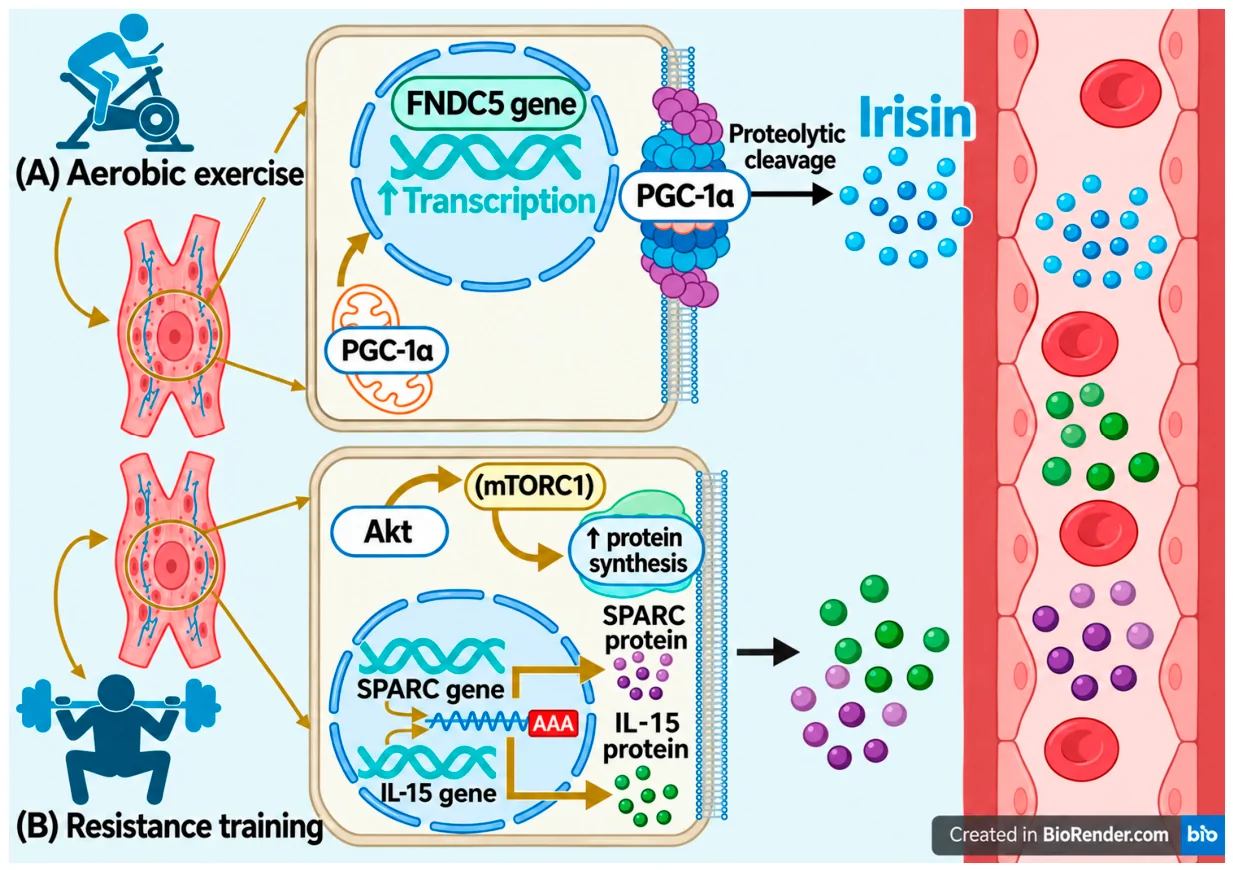
Exercise-induced signaling pathways involved in skeletal muscle adaptation and myokine release. Aerobic exercise leads to the activation of the transcription of FNDC5 dependent on PGC-1α, which causes the further cleavage of FNDC5 and release of irisin into the blood stream. Resistance exercise, in turn, triggers anabolic signaling and activates the Akt/mTORC1 pathway for stimulating muscle protein synthesis. Meanwhile, exercise-induced transcriptional response facilitates the chain of events in terms of the secretion of myokines (e.g., IL-15, SPARC).

Aerobic and resistance exercise therefore activate complementary molecular programs within skeletal muscle, linking exercise-induced metabolic and anabolic signaling with the production of myokines involved in muscle–brain communication, as illustrated in Figure 2 [36,38,64].

High-intensity interval training (HIIT) and high-intensity circuit training (HICT) are time-efficient modalities that may improve metabolic flexibility and recruit fast motor units through repeated short bursts of activity. These modalities can induce acute increases in exercise-responsive factors such as IL-6, IL-15, and vascular endothelial growth factor (VEGF). However, their application may be limited in older adults and individuals with established physical frailty because of the potential risks of musculoskeletal injury and cardiovascular stress. The substantial energy demands of HIIT may also result in transient reductions in cognitive performance immediately after exercise in some older adults. Therefore, these modalities may not be appropriate as first-line interventions for patients with advanced sarcopenia without careful assessment, supervision, and individualized progression [32,33,34].

### 7.2. Pharmacological Modulation of Myokine and Myostatin–Activin Signaling

Myostatin–activin inhibition and replacement of a putatively protective myokine are distinct strategies. MYO-029, trevogrumab, and domagrozumab target myostatin; bimagrumab targets activin type II receptors; ACE-031 is an ActRIIB-Fc ligand trap; and ACE-083 is a locally acting follistatin-based ligand trap. They should not be grouped as myokine mimetics. Clinical investigations have frequently found muscle growth without corresponding functional improvement [64,83,84].

Recombinant myokines and receptor-directed approaches form a separate research category. The astrocytic irisin mechanism discussed in Section 5.1 provides preclinical target evidence, not evidence of efficacy in patients [57,83,85].

Formulation strategies and small-molecule agonists should be described as proposed approaches unless supported by agent-specific studies demonstrating pharmacokinetics, brain exposure, selectivity, and biological activity. Gene transfer and extracellular-vesicle delivery are considered separately in Section 7.3 and Section 7.4.

### 7.3. Gene Therapy

Gene transfer is distinct from pharmacological myokine replacement. Follistatin gene transfer has undergone small early-phase investigations in neuromuscular disease, which do not establish efficacy for age-related sarcopenia or cognitive decline [57,83]. Peripheral irisin expression using a liver-directed viral vector has been investigated in mouse models, as discussed in Section 8.2 [57,83]. Neither strategy should be presented as an established treatment for disruption of the human muscle–brain axis [57,83].

**Figure 3:**
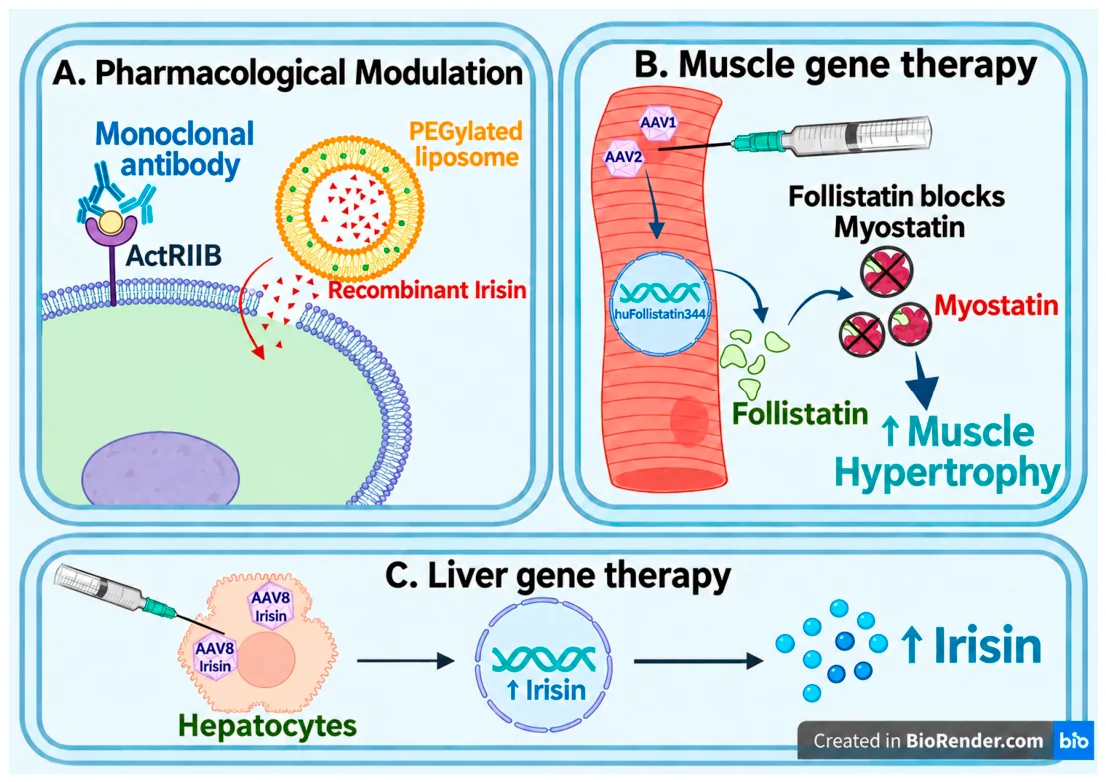
Experimental therapeutic strategies targeting muscle-related signaling. (**A**) Myostatin–activin inhibition and recombinant myokine delivery represent separate modalities; the illustrated irisin formulation is a proposed delivery concept. (**B**) Follistatin gene transfer is intended to inhibit myostatin-related signaling. (**C**) Liver-directed irisin expression has been investigated experimentally. The panels do not imply equivalent evidence, tissue specificity, or demonstrated cognitive benefit in humans.

Taken together, these approaches illustrate two complementary strategies for therapeutic manipulation of the muscle–brain axis: pharmacological modulation of myokine or myostatin signaling, and gene-based approaches designed to provide sustained expression of muscle-related therapeutic factors [35]. Representative experimental strategies are summarized in Figure 3.

### 7.4. Extracellular Vesicles

Extracellular vesicles, including exosomes, provide a specialized mechanism of interorgan communication by protecting regulatory proteins, bioactive lipids, muscle-associated microRNAs, and other nucleic acids from enzymatic degradation in the circulation. Heterochronic studies suggest that circulating factors from young mice can promote muscle regeneration and improve cognitive function in aged mice. Experimental depletion of extracellular vesicles attenuates some of these effects, supporting their potential role in systemic communication and tissue homeostasis.

Extracellular-vesicle engineering remains an investigational delivery strategy. Proposals to load muscle-derived vesicles with neuroactive cargo require direct demonstration of vesicle origin, cargo integrity, biodistribution, and target engagement. Detection of a vesicle-associated signal in the brain should not by itself be interpreted as delivery of biologically active cargo to neurons [34,36].

### 7.5. Precision Medicine

Substantial interindividual variation in the functional responses of older adults to exercise and experimental therapies supports a transition toward a precision medicine approach to the muscle–brain axis. Genetic factors may influence this crosstalk. For example, the apolipoprotein E ε4/ε4 (*APOE* ε4/ε4) genotype alters microglial lipid metabolism, promoting lipid droplet accumulation and the release of neurotoxic factors associated with tau phosphorylation. It may also compromise blood–brain barrier integrity and modify the response to exercise. Similarly, the Val66Met polymorphism in the *BDNF* gene alters activity-dependent BDNF secretion, potentially affecting hippocampal synaptic connectivity and the rate of cognitive decline. Using systems biology and machine-learning methods, precision medicine seeks to identify individual multi-omics signatures by integrating genomic, proteomic, and metabolomic data, including circulating metabolites such as niacin and sphingolipids. These signatures may facilitate the stratification of patients with sarcopenia and the development of individualized exercise prescriptions specifying the volume, intensity, and frequency required to safely maximize neuroprotection and recovery of muscle mass [36,38,64,85].

## 8. Current Challenges and Future Perspectives

Despite advances in characterizing the muscle–brain axis, many of its molecular signaling pathways remain incompletely understood. Translating experimental findings into safe and effective therapies for humans will require addressing substantial methodological, analytical, and clinical limitations [86,87].

### 8.1. Orphan Receptors and Biological Mechanisms Yet to Be Clarified

For many candidate myokines, the relevant receptors and target cells in the central nervous system remain insufficiently characterized. Functional evidence for irisin–αVβ5 signaling in astrocytes is described in Section 5.1. Receptor expression alone does not establish ligand binding, receptor requirement, or a physiological endocrine effect. Future studies should combine binding measurements with cell-specific loss-of-function and rescue experiments [86,87].

A similar limitation applies to other pleiotropic factors, such as osteonectin (SPARC), whose effects in the brain parenchyma appear to be complex and dependent on the inflammatory context. SPARC has been associated with amyloid clearance in preclinical models; however, abnormal concentrations have also been proposed to compromise the permeability and selectivity of the blood–brain barrier under severe inflammatory conditions, potentially exacerbating neurodegenerative processes [33,38,57,81].

### 8.2. The Enigma of Transport Across the Blood–Brain Barrier

Transport across the blood–brain barrier requires molecule-specific evidence. In mice, liver-directed irisin expression increased irisin protein in perfused brain tissue without increasing brain irisin mRNA, supporting peripheral-to-central transfer under those experimental conditions. This does not establish the endothelial transport route or physiological delivery from human skeletal muscle. Direct transport, local neural production, signaling at the vascular interface, and indirect systemic effects should therefore be tested separately rather than treated as interchangeable explanations [33,34,36,48].

### 8.3. Absence of Specific Biomarkers and Analytical Inconsistencies

The clinical utility of myokines as diagnostic biomarkers is limited by their lack of tissue specificity and the absence of reliable muscle-specific markers in the peripheral circulation. Important trophic factors such as BDNF and irisin are not produced exclusively by skeletal muscle; they may also originate from adipose tissue, the liver, immune cells, and neural tissue. Consequently, determining whether changes in their circulating concentrations reflect skeletal muscle adaptations or responses originating in other tissues remains difficult [18,88]. Additional inconsistencies arise from the limited specificity of some commercially available immunoassays, including reported cross-reactivity and positive signals in both FNDC5-knockout and wild-type mice. Discrepancies between serum irisin concentrations measured using commercial enzyme-linked immunosorbent assays and tandem mass spectrometry highlight the need to standardize analytical methods before these molecules can be established as reliable clinical biomarkers [33,36,57,85].

### 8.4. Limited Clinical Evidence in Humans and the Translational Gap

Most mechanistic evidence is derived from cellular or animal systems. Human neural cultures permit controlled pathway testing but do not reproduce an intact circulation or blood–brain barrier. Rodent genetic experiments can establish effects within a model, yet global deletion does not isolate skeletal muscle as the relevant tissue source. Human observational associations remain vulnerable to confounding and reverse causation. Translation therefore requires source-specific studies, relevant exposure measurements, and intervention designs that assess both muscle and cognitive outcomes [47,57,85].

### 8.5. Need for Multi-Omics Integration and Systems Biology

Multi-omics findings can generate hypotheses, but statistical attenuation should not be equated with biological mediation. In a cross-sectional Health ABC analysis, four metabolites together attenuated the association between intermuscular fat and cognitive processing speed by 41%. The design could not establish temporal ordering or causality [47,57,85]. Prospective replication and targeted experiments are needed before these findings can support biomarker or therapeutic use.

The study also identified 1-methylnicotinamide (1-MNA), a niacin-derived muscle-associated metabolite, as a potential mediator of muscle–brain communication. Experimental evidence suggests that 1-MNA may attenuate neuroinflammation induced by lipopolysaccharides or amyloid-β peptides by modulating microglial activation and suppressing NF-κB signaling. These findings highlight the importance of examining interacting networks of metabolites and proteins rather than individual molecules in isolation [36,64,89].

### 8.6. Artificial Intelligence in Clinical Diagnosis and Monitoring

Artificial intelligence (AI) and machine learning (ML) are emerging as potentially valuable tools for analyzing the large volumes of multi-omics and clinical data generated in studies of the muscle–brain axis. Machine-learning algorithms can integrate heterogeneous variables and may help predict individual trajectories of cognitive decline and muscle loss. Models incorporating data from wearable motion sensors may also support the analysis of gait and movement patterns. Clinically, AI-assisted analysis of wearable-sensor data could facilitate early, noninvasive screening for sarcopenia and associated cognitive decline by detecting subtle changes in balance, gait, and walking speed that may not be captured by conventional clinical assessments [36,64].

### 8.7. Personalized Medicine and Individual Stratification

Future interventions aimed at preserving muscle and cognitive health should be guided by the principles of personalized and precision geriatric medicine. Biological responses within the muscle–brain axis vary substantially among individuals according to factors such as sex, insulin resistance, and genetic background [19,90]. For example, the apolipoprotein E ε4 (*APOE* ε4) allele, an important risk factor for Alzheimer’s disease, alters lipid metabolism in brain microglia. This alteration promotes the accumulation of lipid droplets, exacerbates inflammation, and may accelerate tau phosphorylation. The *APOE* ε4 allele may also compromise blood–brain barrier integrity, potentially altering the transport and central bioavailability of exercise-induced protective myokines. Characterizing these individual determinants through multi-omics approaches may help clinicians develop individualized exercise prescriptions and combination therapies aimed at safely improving neuromuscular and cognitive resilience [36,38,64,89].

Near-term priorities are analytical validation of candidate assays, repeated sampling in well-characterized cohorts, and prespecified assessment of muscle and cognitive outcomes in intervention studies. Source attribution and central target engagement require mechanistic substudy designs. Biomarker-guided exercise selection, receptor-directed therapy, and engineered-vesicle delivery should remain investigational until independently validated. Computational models should be evaluated in external cohorts and against existing clinical assessments before being used to guide care.

## 9. Conclusions

Evidence increasingly links muscle health with brain health during ageing. Sarcopenia, physical inactivity, chronic inflammation, and mitochondrial dysfunction frequently coexist with cognitive impairment and neurodegenerative disease. Muscle-derived factors may participate in this relationship, but their contribution remains uncertain. In humans, it has not yet been established whether individual myokines reach the brain in biologically relevant concentrations or whether they act mainly through peripheral metabolic, vascular, and inflammatory pathways.

This distinction is important because much of the mechanistic evidence comes from cellular and animal models, whereas human studies remain largely observational. Uncertainty regarding tissue origin, receptor specificity, blood–brain barrier transport, and measurement of circulating myokines also makes it difficult to determine whether changes in these molecules are causes, consequences, or compensatory responses to ageing and disease.

Exercise remains the most clearly supported intervention for preserving muscle function and is also associated with better cognitive outcomes. Whether these benefits can be reproduced by targeting individual myokines is not yet known. Progress in this field will depend on human studies that combine standardized biomarker measurements with longitudinal assessments of muscle function, cognition, and brain structure. Such studies are needed before the muscle–brain axis can be translated from a biologically plausible model into a reliable therapeutic target.

## Figures and Tables

**Table 1:** Neuroprotective myokines involved in muscle–brain crosstalk.

Myokine	Main Mechanism of Action	Effects on the Brain	Main Potential Benefit
Irisin	Activates αV/β5 integrins and downstream signaling pathways associated with BDNF expression [56].	Enhances synaptic plasticity and hippocampal neurogenesis [56,57].	Potential improvement in memory and cognitive function [56,57].
Cathepsin B	Promotes BDNF and DCX expression in the hippocampus [48,58].	Stimulates adult neurogenesis and may contribute to β-amyloid clearance [34,48].	Potential improvement in memory and learning [34,38].
BDNF	Binds to TrkB and activates PI3K/Akt and MAPK/ERK signaling [33,56].	Promotes neuronal survival and synaptic plasticity [33,34,56].	Supports memory, learning, and hippocampal function [33,34,59].
LIF	Signals through the LIFRβ/gp130 receptor complex and Akt-associated pathways [38,60,61].	Supports neurogenesis and may reduce neuronal apoptosis [38,60,61].	Potential protection against neuronal injury [38,60,61].

**Table 2:** Anti-inflammatory and metabolic myokines involved in muscle–brain crosstalk.

Functional Category	Myokine	Main Mechanism of Action	Main Potential Benefit
Anti-inflammatory	IL-10	Suppresses pro-inflammatory mediators, including TNF-α and IL-6, and modulates microglial activation [29,59].	Attenuation of neuroinflammation and potential neuronal protection [29,59].
Anti-inflammatory	IL-15	Modulates inflammatory responses, apoptosis, muscle metabolism, and insulin sensitivity [38,62,63].	Supports muscle homeostasis and immune regulation and may modulate neuroinflammation [38,62,63].
Anti-inflammatory/reparative	SPARC	Participates in tissue remodeling and has been associated with β-amyloid clearance [25,38,60,61].	Potential contribution to tissue repair and neuroprotection [25,38,60,61].
Metabolic	FGF21	Regulates mitochondrial function and cellular energy metabolism [5,33,38,48,61].	May reduce oxidative stress and support neuronal and cerebrovascular protection [5,33,38,48,61].
Metabolic	BAIBA	Activates AMPK and PI3K/Akt signaling [58,60].	Supports energy homeostasis and antioxidant defense [58,60].
Metabolic/neurovascular	Apelin	Activates APJ-mediated PI3K/Akt/mTOR signaling [33,58,61,64].	May promote angiogenesis, cerebral perfusion, and neuronal survival [33,58,61,64].

## Data Availability

No new data were created or analyzed in this study. Data sharing is not applicable to this article.

## Abbreviations

Aβ	Amyloid-beta
AD	Alzheimer’s disease
AI	Artificial intelligence
ALS	Amyotrophic lateral sclerosis
AMP	Adenosine monophosphate
AMPK	AMP-activated protein kinase
APJ	Apelin receptor
APLNR	Apelin receptor
APOE	Apolipoprotein E
APP	Amyloid precursor protein
ATP	Adenosine triphosphate
BAIBA	β-aminoisobutyric acid
BBB	Blood–brain barrier
BDNF	Brain-derived neurotrophic factor
CAG	Cytosine–adenine–guanine
cAMP	Cyclic adenosine monophosphate
CNS	Central nervous system
CREB	cAMP response element-binding protein
CRP	C-reactive protein
CTSB	Cathepsin B
DAMPs	Damage-associated molecular patterns
DCX	Doublecortin
DHEA-S	Dehydroepiandrosterone sulfate
DNA	Deoxyribonucleic acid
E3	E3 ubiquitin ligase
EAE	Experimental autoimmune encephalomyelitis
ERK	Extracellular signal-regulated kinase
FGF21	Fibroblast growth factor 21
FGFR1c	Fibroblast growth factor receptor 1c
FNDC5	Fibronectin type III domain-containing protein 5
FS344	Follistatin 344 isoform
GDF-8	Growth differentiation factor 8
GH	Growth hormone
gp130	Glycoprotein 130
HD	Huntington’s disease
HDAC4	Histone deacetylase 4
HICT	High-intensity circuit training
HIIT	High-intensity interval training
HPA	Hypothalamic–pituitary–adrenal
HTT	Huntingtin gene
IGF-1	Insulin-like growth factor 1
IκB	Inhibitor of NF-κB
IKK	IκB kinase
IL-1β	Interleukin-1 beta
IL-2	Interleukin-2
IL-6	Interleukin-6
IL-10	Interleukin-10
IL-15	Interleukin-15
IL-15Rα	Interleukin-15 receptor alpha
IL-2/15Rβ	Interleukin-2/15 receptor beta
IRS-1	Insulin receptor substrate 1
JAK	Janus kinase
Kir2.1	Inward rectifier potassium channel 2.1
LIF	Leukemia inhibitory factor
LIFRβ	Leukemia inhibitory factor receptor beta
LPC	Lysophosphatidylcholine
LTP	Long-term potentiation
MAPK	Mitogen-activated protein kinase
MAPK/ERK	Mitogen-activated protein kinase/extracellular signal-regulated kinase
MCI	Mild cognitive impairment
ML	Machine learning
mPTP	Mitochondrial permeability transition pore
mTOR	Mechanistic target of rapamycin
mTORC1	Mechanistic target of rapamycin complex 1
MuRF1	Muscle RING-finger protein 1
MYO-029	Myostatin inhibitor/investigational compound
NAD+	Nicotinamide adenine dinucleotide
NAMPT	Nicotinamide phosphoribosyltransferase
NEP	Neprilysin
NF-κB	Nuclear factor kappa B
NLRP3	NLR family pyrin domain containing 3
NRTN	Neurturin
OR	Odds ratio
PD	Parkinson’s disease
PGC-1α	Peroxisome proliferator-activated receptor gamma coactivator 1-alpha
PI3K	Phosphoinositide 3-kinase
PKA	Protein kinase A
PPARγ	Peroxisome proliferator-activated receptor gamma
ROS	Reactive oxygen species
ROM	Range of motion
SIRT1	Sirtuin 1
sNfL	Serum neurofilament light chain
SOD	Superoxide dismutase
SPARC	Secreted protein acidic and rich in cysteine
STAT3	Signal transducer and activator of transcription 3
TFAM	Mitochondrial transcription factor A
TFEB	Transcription factor EB
TGF-β	Transforming growth factor beta
TLR9	Toll-like receptor 9
TNF-α	Tumor necrosis factor alpha
TrkB	Tropomyosin receptor kinase B
UPS	Ubiquitin–proteasome system
VEGF	Vascular endothelial growth factor
αV/β5	Integrin alpha V/beta 5
αVβ5	Alpha V beta 5 integrin
β-Klotho	Beta-Klotho
βγ	Beta-gamma receptor complex
cAMP/PKA/CREB	cAMP/PKA/CREB signaling pathway
AMPK-SIRT1	AMPK–SIRT1 signaling axis
PGC-1α/FNDC5	PGC-1α/FNDC5 pathway
p53	Tumor protein p53
LPC 18:1	Lysophosphatidylcholine 18:1
LPC 18:2	Lysophosphatidylcholine 18:2
1-MNA	1-Methylnicotinamide
AAV8	Adeno-associated virus serotype 8
rAAV1	Recombinant adeno-associated virus serotype 1
CMV	Cytomegalovirus promoter
AAV8-irisin-FLAG	AAV8 vector expressing irisin with FLAG tag
AdFNDC5	Adenoviral FNDC5 vector/construct
ACE-031	ActRIIB-Fc fusion protein
ACE-083	Follistatin-based ligand trap
ActRIIB-Fc	Activin receptor type IIB-Fc
HTT	Huntingtin
ClC-1	Chloride channel 1
Kir2.1	Potassium channel 2.1
CAG	CAG trinucleotide
APP/PS1	Amyloid precursor protein/presenilin 1
5xFAD	Five familial Alzheimer’s disease mutations
